# Elman Neural Network-Based Direct Lift Automatic Carrier Landing Nonsingular Terminal Sliding Mode Fault-Tolerant Control System Design

**DOI:** 10.1155/2023/3560441

**Published:** 2023-01-09

**Authors:** Qilong Wu, Qidan Zhu, Shuai Han

**Affiliations:** College of Intelligent Systems Science and Engineering, Harbin Engineering University, Harbin, Heilongjiang 150001, China

## Abstract

The purpose of this paper is to develop the control system using the Elman neural network (ENN) and nonsingular terminal sliding mode control (NTSMC) to improve the automatic landing capability of carrier-based aircraft based on direct lift control (DLC) when subjected to carrier air-wake disturbance and actuator failure. First, the carrier-based aircraft landing model is derived. Then, the NTSMC is proposed to ensure the system's robustness and achieve accurate trajectory tracking performance in a finite time. Due to the inclusion of nonsingularity in NTSMC, the steady-state response of the control system can be effectively improved. In addition, the ENN is derived using an adaptive learning algorithm to approximate the actuator faults and system uncertainties. To further ensure the accurate tracking of the ideal glide path by the carrier-based aircraft, the NTSMC system using an ENN estimator is proposed. Finally, this method is tested by adding different types of actuator failures. The simulation results show that the designed longitudinal fault-tolerant carrier landing system has strong robustness and fault-tolerant ability and improves the accuracy of carrier-based aircraft landing trajectory tracking.

## 1. Introduction

Landing control has been an important research topic since the birth of carrier-based aircraft. The technical difficulty of landing control is adjusting the performance of carrier-based aircraft to land in a very small safe area within the limited time and space of the landing process. Normally, the conventional automatic carrier landing system (ACLS) can guarantee a successful landing [1–3]. However, in air combat, aircrafts are susceptible to combat damage, which can lead to component failures such as actuator, sensor, engine, or system, resulting in flight performance degradation or instability [4]. If correct and effective fault-tolerant control is not carried out in time, it will largely cause loss of personnel and property, and the consequences will be serious.

To improve the ability of carrier-based aircraft to deal with various faults, it is necessary to adopt a more effective control strategy, that is, fault-tolerant control [5]. Fault-tolerant control can make the aircraft continue to fly or land safely in case of failure and performance degradation, avoiding air accidents. After decades of development, fault-tolerant control technology based on modern control theory has made some achievements. However, with the increase in the complexity of the control object and the difficulty of flight tasks, there is an urgent need to introduce fault-tolerant control methods based on nonlinear control, intelligent control, and other advanced control technologies. For the actuator failure of a nonlinear flexible wing system, the new adaptive fault-tolerant boundary control scheme is proposed that can be automatically updated to compensate for the system's actuator failure [6]. However, the adaptive controller is difficult to adapt to changes quickly. Especially when the characteristics of the nonlinear model change significantly, the controller parameters often need to be adjusted in time. The adaptive nonlinear sliding mode control combined with the baseline nonlinear dynamic inverse (NDI) controller applied to unmanned aerial vehicles (UAVs) has shown good performance [7, 8]. However, the NDI control method is highly dependent on the model accuracy, and the control performance of the dynamic inverse controller will drop sharply when the model data are inaccurate. The adaptive fault-tolerant *H*-infinity tracking controller is designed for the flight control system with actuator failure, which improves the system's dynamic performance and robust performance [9, 10]. However, this approach needs to be simplified in dealing with high-gain systems and designing high-order controllers. The neural network has approximate nonlinear functions and online learning capability, providing a fast mechanism for the aircraft control system to adapt to unknown actuator failures, structural damage, and wind disturbances. For the air-breathing hypersonic vehicle, the fault-tolerant control method combining a radial bias function neural network (RBFNN) and sliding mode method is proposed, which solves the problems of actuator partial failure fault and bias fault [11]. Ismail et al. [12, 13] presented the fault-tolerant control system combining the sliding mode and RBF neural network applied to the automatic landing of conventional fighter aircraft, which can solve the failure problems of fighter aircraft in encountering strong winds and rudder stuck without fault diagnosis. The aircraft control system is designed by using a recursive wavelet Elman neural network to ensure the successful automatic landing of commercial aircraft when it suffers from severe wind disturbances and failures [14]. The most significant advantage of the recursive wavelet Elman neural network method compared to traditional neural networks is its real-time learning ability. Design methods based on reinforcement learning and neural networks have been widely used [15, 16]. In [17], significant progress was made by applying the IFT algorithm to solve optimization problems.

Sliding mode control has been widely used [18]. Conventional sliding mode control cannot make the system state reach the equilibrium point in a limited time. Compared with the conventional SMC with a linear sliding surface, the NTSMC offers some superior properties, such as faster tracking response, finite time convergence, and higher control precision [19]. Referring to the previous research results, this article proposes the fault-tolerant control method combining ENN and NTSMC (ENN-NTSMC). Because of the context neurons and local recurrent connections between the context layer and the hidden layer, it has certain dynamic advantages over static NNs [20]. The application of the ENN estimator can solve the requirement of NTSMC for uncertainty [21–23]. Furthermore, the adaptive learning algorithms of the ENN are derived using the Lyapunov stability theorem. The ENN was originally applied to speech processing [24]. In addition, the ENN has different applications in other fields, such as aircraft engine systems [25], Internet traffic prediction [26], multisensor data fusion [27], indoor temperature prediction [28], and hydraulic servo systems [29]. A typical ENN cannot accurately approximate higher-order dynamic systems, and its convergence rate is usually slow, which is unsuitable for specific time-critical applications. Therefore, the improved ENN is proposed to overcome these problems in this article. The improved ENN increases the internal self-connected signals of the nodes in the context layer, which speeds up the convergence rate and can better approximate the unknown functions.

In addition, for research on aircraft motion, the literature [30] proposed a new test platform system for different vertical take-off and landing multirotor UAVs that can achieve unrestricted aircraft motion along all axes. The literature [31] introduces direct lift control (DLC) into the flight control system. Simulations and flight tests show that accurate flight path tracking can be achieved by introducing DLC. There are few changes in the way the aircraft is maneuvered, with approach power controlled by the throttle and altitude deviation controlled by the elevator. However, suppose only the elevator is used as the only altitude control rudder surface. In that case, it is difficult to accurately track the ideal slide path in air-wake turbulence, thus proposing the DLC scheme for carrier-based aircraft landing [32]. This technique uses the elevator and flaps to form a pair of control rudder surfaces that can balance the pitch moment and directly control the forces acting on the aircraft. The lift is directly generated by the flaps rather than indirectly generated by the angle of attack (AOA) and pitch angle rate, so the coupling of trajectory motion and attitude motion can be eliminated.

Referring to the previous research results, the fault-tolerant control method based on NTSMC and ENN is proposed to optimize the performance of the longitudinal automatic carrier landing system based on direct lift control (DLC-ACLS) when the aircraft encounters elevator and engine failures. This article is motivated by the tracking control of the DLC-ACLS with the effect of actuator failure and disturbances. The NTSMC scheme based on the ENN is developed for tracking the reference trajectory. The main contributions of this article are as follows:

1. The proposed direct lift control strategy can directly change the lift force through the flaps, eliminating the coupling of trajectory motion and attitude motion, which is very beneficial to the precise control of the automatic landing trajectory.
2. This article designs an intelligent control scheme for the DLC-ACLS using the ENN-NTSMC to improve the performance of aircraft ACLS. The proposed control scheme will deal with uncertainties, actuator failure, and disturbances to achieve a safe landing.
3. The Lyapunov stability theorem and Barbalat's lemma ensure that the system is globally asymptotically stable and the errors of the state variables can converge to 0.

The structure of this article is as follows: Section 2 describes the landing problem of carrier-based aircraft, including the nonlinear model of the carrier-based aircraft, the carrier motion model, and the carrier air-wake model; Section 3 designs the fault-tolerant control method based on the combination of NTSMC and ENN, and Section 4 applies it to the longitudinal DLC-ACLS; Section 5 gives the design of the simulation experiment and the simulation results, which verifies the effectiveness of the design method; and Section 6 gives the conclusion of this paper.

## 2. Landing Model Building

**Figure 1:**
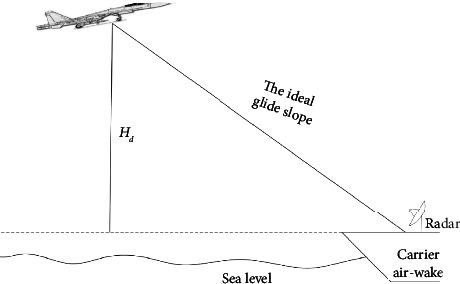
Final carrier landing phase.

During the landing process, it is necessary to control the aircraft's speed to remain constant to make the aircraft fly along the ideal glide path. The final landing stage is shown in Figure 1. This section describes the longitudinal aircraft model, the carrier air-wake model, and the deck motion model.

**Assumption 1 .**

The Earth is regarded as a plane, and the aircraft is a rigid body with a symmetrical plane flying in a vertical plane.

**Assumption 2 .**

Uncertainties and faults are unknown and bounded, and there exist positive constants *d*_1_ and *d*_2_ such that |*f*(*x*)| ≤ *d*_1_ and |*δ*| ≤ *d*_2_.

**Assumption 3 .**

The desired value is bounded, and their derivatives are bounded. Furthermore, all system states can be measured.

### 2.1. Establishing the Longitudinal Model of Carrier Aircraft Landing with Air-Wake Disturbance

**Figure 2:**
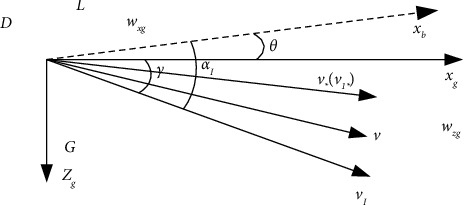
Analysis of the forces during landing.

In a nonstationary atmosphere, the force analysis of the aircraft is shown in Figure 2.

Projecting the gust velocity vector *v*_*w*_ into the horizontal and vertical directions, the horizontal component *w*_*xg*_ and the vertical component *w*_*zg*_ are obtained. A large number of experimental results show that the airspeed *v* and the AOA are mainly disturbed by *w*_*xg*_ and *w*_*zg*_, respectively.

After decoupling, the longitudinal carrier-based aircraft dynamics equations are presented as follows [33]:

(1) $$\begin{array}{c} \dot{V}=\frac{P\;\cos\;\alpha -D}{m}-g\;\sin\gamma \text{,} \end{array}$$

(2) $$\begin{array}{c} \dot{\gamma }=\frac{L+P\;\sin\;\alpha }{mV}-\frac{g}{V}\cos\;\gamma \text{,} \end{array}$$

(3) $$\begin{array}{c} \dot{h}=V\;\sin\;\gamma \text{,} \end{array}$$

(4) $$\begin{array}{c} \alpha =q-\dot{\gamma }\text{,} \end{array}$$

(5) $$\begin{array}{c} \dot{\theta }=q\text{,} \end{array}$$

(6) $$\begin{array}{c} \dot{q}=\frac{M}{I_{yy}}\text{,} \end{array}$$

(7) $$\begin{array}{c} D=0.5\rho V^{2}SC_{D}\left(\alpha ,\delta_{e},\delta_{c}\right)\text{,} \end{array}$$

(8) $$\begin{array}{c} L=0.5\rho V^{2}SC_{L}\left(\alpha ,\delta_{e},\delta_{c},\delta_{f}\right)\text{,} \end{array}$$

(9) $$\begin{array}{c} M=0.5\rho V^{2}S\bar{c}C_{M}\left(\alpha ,q,\delta_{e},\delta_{c},\delta_{f}\right)\text{,} \end{array}$$

(10) $$\begin{array}{c} \left\{\begin{array}{c} v_{I}\approx v+w_{xg}\\ \alpha_{I}\approx \alpha +\frac{w_{zg}}{v_{*}} \end{array}\right.\text{,} \end{array}$$

where *V* is the speed, *v*_*I*_ is the inertial speed (ground speed), and *w*_*xg*_ and *w*_*zg*_ are the wind speeds. *γ* is the flight trajectory angle, *α* denotes the AOA, and *α*_*I*_ is the inertial angle of attack. *q* denotes the pitch angular rates, *θ* is the pitch angle, and *δ*_*c*_, *δ*_*e*_, and *δ*_*f*_ are the deflection angles of the canard, elevator, and flap, respectively. *C*_*L*_, *C*_*D*_, and *C*_*M*_ are the coefficients of lift, drag, and pitch moment, respectively. *h* is the altitude. *ρ* is the air density, *I*_*yy*_ is the pitch moment of inertia. *P* is the engine thrust. *M* is the pitch moment.

Based on the longitudinal model under windy conditions, the longitudinal small disturbance equation can be obtained as follows:

(11) $$\begin{array}{c} \left\{\begin{array}{c} x_{w}=Ax_{w}+Bu+Ew\text{,}\\ y_{w}=Cx_{w}+Du+Fw\text{,} \end{array}\right. \end{array}$$

where $x_{w}={\left[\begin{array}{ccccc} ∆v_{I} & ∆\alpha_{I} & ∆q & ∆\theta & ∆h \end{array}\right]}^{T}$, $y_{w}={\left[\begin{array}{ccccccc} ∆v_{I} & ∆\alpha_{I} & ∆q & ∆\theta & ∆h & \left(∆n_{z}/v_{*}\right) & ∆\gamma \end{array}\right]}^{T}$, $w={\left[\begin{array}{cc} w_{xg} & w_{zg} \end{array}\right]}^{T}$, and $u={\left[\begin{array}{cccc} ∆\delta_{e} & ∆\delta_{c} & ∆\delta_{p} & ∆\delta_{f} \end{array}\right]}^{T}$.

### 2.2. Air-Wake Model

Because the landing environment is highly challenging, the carrier air-wake is modeled using the US military standard MIL-F-8785C [34]. The US military specification MIL-F-8785C decomposes the aircraft carrier air-wake into three components in perpendicular directions: the horizontal longitudinal component *u*, the horizontal lateral component *v*, and the vertical component *w*. The calculation formula is as follows:

(12) $$\begin{array}{c} \left\{\begin{array}{c} u=u_{1}+u_{2}+u_{3}+u_{4}\text{,}\\ v=v_{1}+v_{4}\text{,}\\ w=w_{1}+w_{2}+w_{3}+w_{4}\text{.} \end{array}\right. \end{array}$$

**Figure 3:**
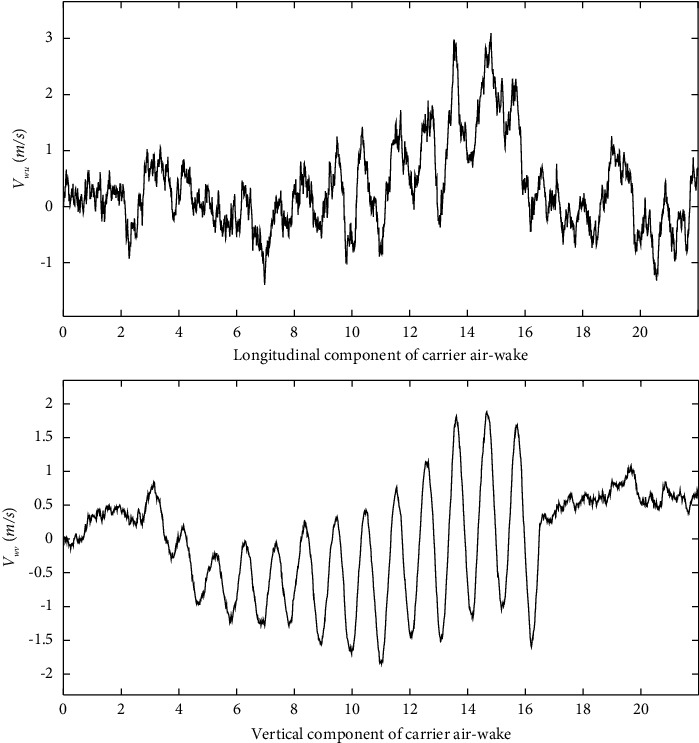
Component of the carrier air-wake.

According to equation (12), the simulation curve is shown in Figure 3.

Through the analysis, the following two conclusions can be drawn:

1. The smaller the distance between the carrier aircraft and the deck, the greater the disturbance of the ship wake
2. The effect of the lateral disturbance component is not negligible when the carrier-based aircraft is near the end of the deck

### 2.3. Carrier Motion Model

When an aircraft carrier is sailing in waves, the movement of the hull has an adverse effect on landing. This article refers to the US AD report and uses a combination of sine functions to simulate the movement of the aircraft carrier at a typical speed. The formula is as follows.

Pitch motion:

(13) $$\begin{array}{c} \theta_{c}=0.5\;\sin\;\left(0.6t\right)+0.3\;\sin\;\left(0.63t\right)+0.25\text{,} \end{array}$$

where *θ*_*c*_ is the change in the pitch angle generated by the pitching motion of the ship.

Heave motion:

(14) $$\begin{array}{c} h_{c}=1.22\;\sin\;\left(0.6t\right)+0.3048\;\sin\;\left(0.2t\right)\text{,} \end{array}$$

where *h*_c_ is the height change caused by the vertical undulating motion of the ship.

## 3. Fault-Tolerant Control System Design Based on ENN-NTSMC

**Figure 4:**
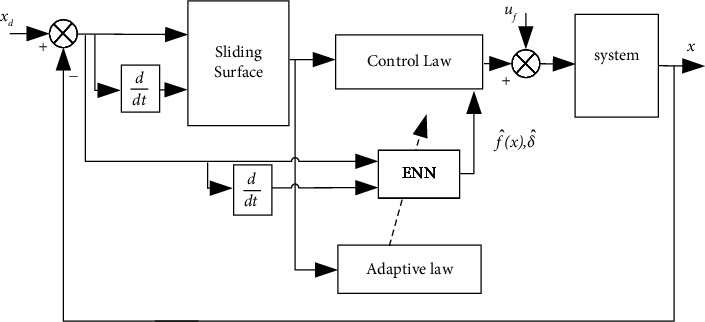
Fault-tolerant controller structure block diagram.

**Figure 5:**
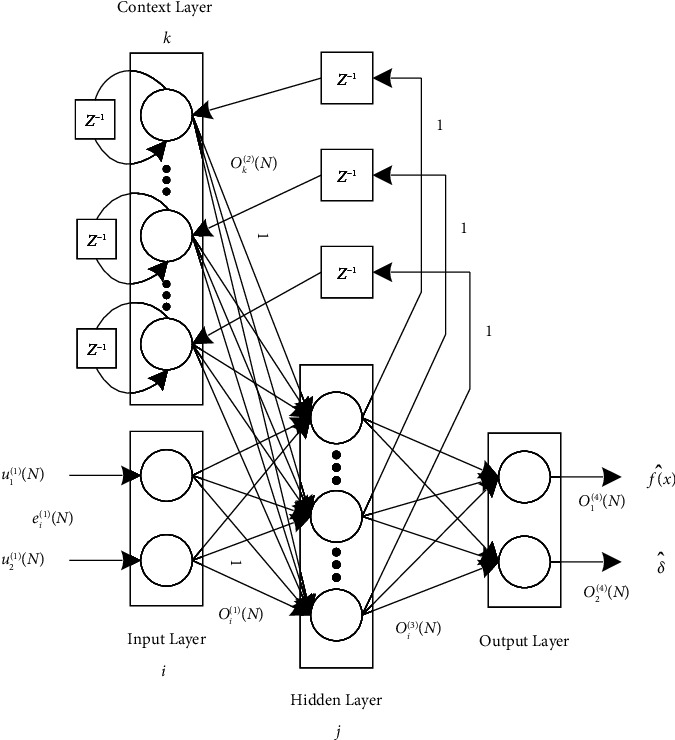
Structure of the Elman neural network.

The structure of the fault-tolerant controller designed is shown in Figure 4. The ENN estimator directly estimates the system fault function *δ* and uncertainty term *f*(*x*). The neural network weights are adjusted by the adaptive law [35], and the controller is the NTSMC. The structure of the ENN consists of an input layer, a hidden layer, a context layer, and an output layer, as shown in Figure 5.

### 3.1. ENN Estimator

The following is the neural network estimator design.

(1) Input layer: the input value of the input layer is the position tracking error *e* and its differential $\dot{e}$. The input and output of the node can be defined as follows:
  (15) $$\begin{array}{c} \left\{\begin{array}{c} u_{1}^{\left(1\right)}\left(N\right)=e,\\ u_{1}^{\left(1\right)}\left(N\right)=\dot{e,} \end{array}\right.\\ o_{i}^{\left(1\right)}\left(N\right)=u_{i}^{\left(1\right)},i=1,2\text{,} \end{array}$$
where *i* is the number of neurons and *N* is the number of iterations.
(2) Context layer: in the context layer, the nodes are represented as
  (16) $$\begin{array}{c} u_{k}^{\left(2\right)}=\beta o_{k}^{\left(2\right)}\left(N-1\right)+o_{j}^{\left(3\right)}\left(N-1\right)j=1,2,\ldots ,9k=1,2,\ldots ,9\text{,} \end{array}$$
where *u*_*k*_^(2)^ and *o*_*k*_^(2)^ are the input and output of the *k*th node in the layer, respectively. 0 ≤ *β* < 1 is the self-connected feedback gain. *o*_*j*_^(3)^ is the output of the hidden layer. *j* and *k* are the number of neurons in the context and hidden layers, respectively.
(3) Hidden layer: in the hidden layer, the nodes are defined by
  (17) $$\begin{array}{c} o_{j}^{\left(3\right)}=\frac{1}{1+e^{\theta_{j}}}\text{,}\\ \theta_{j}=\overset{2}{\underset{i=1}{\sum }}o_{i}^{\left(1\right)}+\overset{9}{\underset{k=1}{\sum }}o_{k}^{\left(2\right)}\text{,} \end{array}$$
where *θ*_*j*_ and *o*_*j*_^(3)^ are the input and output of the *j*th node in the hidden layer, respectively. *θ*_*j*_ is the sum of the output values of the input layer and the context layer. For the convenience of calculation, the connection weights except for the hidden neuron to the output neuron are set as one.
(4) Output layer: in the output layer, the input and output of the node are represented as
  (18) $$\begin{array}{c} u_{1}^{\left(4\right)}=\overset{9}{\underset{j=1}{\sum }}o_{j}^{\left(3\right)}w_{j}^{3}\text{,}\\ u_{2}^{\left(4\right)}=\overset{9}{\underset{j=1}{\sum }}o_{j}^{\left(3\right)}v_{j}^{3}\text{,}\\ o_{1}^{\left(4\right)}=u_{1}^{\left(4\right)}\text{,}\\ o_{2}^{\left(4\right)}=u_{2}^{\left(4\right)}\text{,} \end{array}$$
where *w*_*j*_^3^ and *v*_*j*_^3^ are the connection weights between the hidden layer and the output layer, respectively. *o*_1_^(4)^ and *o*_2_^(4)^ are the output layers used to estimate the system failure function *δ* and the uncertainty term *f*(*x*), respectively.

### 3.2. ENN-NTSMC System

We consider the second-order nonlinear system in the fault state as follows:

(19) $$\begin{array}{c} \ddot{x}=f\left(x\right)+bu+\delta \text{,} \end{array}$$

where *b* ≠ 0, *δ*=*bu*_*f*_ is the system fault, and *u*_*f*_ is the actuator fault.

Defining the error signal

(20) $$\begin{array}{c} \left\{\begin{array}{c} e=x-x_{d}\text{,}\\ \dot{e}=\dot{x}-{\dot{x}}_{d}\text{,}\\ \ddot{e}=\ddot{x}-{\ddot{x}}_{d.} \end{array}\right. \end{array}$$

We take the sliding mode function as

(21) $$\begin{array}{c} s=e+\frac{1}{\beta }{\dot{e}}^{\left(p/q\right)}\text{,} \end{array}$$

where *β* is a designed positive constant and *p* and *q* are both positive odd integers that should satisfy the following condition: *p* > *q*.

The derivative of the sliding mode function is

(22) $$\begin{array}{c} \dot{s}=\dot{e}+\frac{p}{\beta q}{\dot{e}}^{\left(p/q\right)-1}\ddot{e}\\ =\dot{e}+\frac{p}{\beta q}{\dot{e}}^{\left(p/q\right)-1}\left(\ddot{x}-{\ddot{x}}_{d}\right)\\ =\dot{e}+\frac{p}{\beta q}{\dot{e}}^{\left(p/q\right)-1}\left(f\left(x\right)+bu+\delta -{\ddot{x}}_{d}\right)\text{.} \end{array}$$

To ensure the asymptotic stability of the second-order control system, we define the control input *u* as

(23) $$\begin{array}{c} u=b^{-1}\left(-\frac{\beta q}{p}{\dot{e}}^{2-\left(p/q\right)}-\hat{f}\left(x\right)+{\ddot{x}}_{d}-hsgn\left(s\right)-\hat{\delta }\right)\text{.} \end{array}$$

Using the ENN estimator to directly estimate the system fault function *δ* and the uncertainty term (*x*), the output of the ENN is

(24) $$\begin{array}{c} o_{1}^{\left(4\right)}=\hat{f}\left(x\right)={\hat{W}}^{T}\phi \text{,}\\ o_{2}^{\left(4\right)}=\hat{\delta }={\hat{V}}^{T}\phi \text{,}\\ \phi ={\left[o_{1}^{\left(3\right)}o_{2}^{\left(3\right)}\cdots o_{9}^{\left(3\right)}\right]}^{T}\text{,}\\ \hat{W}=\left[w_{1}^{3}w_{2}^{3}\cdots w_{9}^{3}\right]\text{,}\\ \hat{V}=\left[v_{1}^{3}v_{2}^{3}\cdots v_{9}^{3}\right]\text{,} \end{array}$$

where $\hat{f}\left(x\right)$ and $\hat{\delta }$ are the two outputs of the ENN, *ϕ* is the output vector of the hidden layer *j*th neuron, and $\hat{W}$ and $\hat{V}$ are the weight vectors.

To solve the problem that the system failure function *δ* and the uncertainty term *f*(*x*) cannot be measured, the optimal estimated value is designed as follows:

(25) $$\begin{array}{c} f\left(x\right)=f{\left(x\right)}^{*}+\varepsilon_{1}=W^{*T}\phi +\varepsilon_{f\left(x\right)}\text{,} \end{array}$$

(26) $$\begin{array}{c} \delta =\delta^{*}+\varepsilon_{2}=V^{*T}\phi +\varepsilon_{\delta }\text{,} \end{array}$$

(27) $$\begin{array}{c} \hat{f}\left(x\right)={\hat{W}}^{T}\phi +{\hat{\varepsilon }}_{f\left(x\right)}\text{,} \end{array}$$

(28) $$\begin{array}{c} \hat{\delta }={\hat{V}}^{T}\phi +{\hat{\varepsilon }}_{\delta }\text{.} \end{array}$$

where *W*^*∗T*^*ϕ* and *V*^*∗T*^*ϕ* are the optimal estimates of the system fault function *δ* and the uncertainty term *f*(*x*); *W*^*∗*^ and *V*^*∗*^ are the optimal weights between the hidden and output layers; *ε*_*f*(*x*)_ and *ε*_*δ*_ are the minimum reconstruction errors. Substituting equation (25) into equation (26) and equation (27) into equation (28), the following equations can be obtained:

(29) $$\begin{array}{c} \tilde{f}\left(x\right)=f\left(x\right)-\hat{f}\left(x\right)={\tilde{W}}^{T}\phi +{\tilde{\varepsilon }}_{f\left(x\right)}\text{,}\\ \tilde{\delta }=\delta -\hat{\delta }={\tilde{V}}^{T}\phi +{\tilde{\varepsilon }}_{\delta }\text{,}\\ \tilde{W}=W^{*}-\hat{W}\text{,}\\ \tilde{V}=V^{*}-\hat{V}\text{,}\\ {\tilde{\varepsilon }}_{f\left(x\right)}=\varepsilon_{f\left(x\right)}-{\hat{\varepsilon }}_{f\left(x\right)}\text{,}\\ {\tilde{\varepsilon }}_{\delta }=\varepsilon_{\delta }-{\hat{\varepsilon }}_{\delta }\text{.} \end{array}$$

To prove the stability of the system, the Lyapunov function is defined as

(30) $$\begin{array}{c} L=\frac{1}{2}s^{2}+\frac{1}{2\eta_{1}}{\tilde{W}}^{2}+\frac{1}{2\eta_{2}}{\tilde{V}}^{2}+\frac{1}{2\eta_{3}}{\tilde{\varepsilon }}_{f\left(x\right)}^{2}+\frac{1}{2\eta_{4}}{\tilde{\varepsilon }}_{\delta }^{2}\text{.} \end{array}$$

We take the time derivative of the Lyapunov function and substitute equation (23) into it

(31) $$\begin{array}{c} \dot{L}=s\dot{s}-\frac{1}{\eta_{1}}\tilde{W}\dot{\hat{W}}-\frac{1}{\eta_{2}}\tilde{V}\dot{\hat{V}}-\frac{1}{\eta_{3}}{\tilde{\varepsilon }}_{f\left(x\right)}{\dot{\hat{\varepsilon }}}_{f\left(x\right)}-\frac{1}{\eta_{4}}{\tilde{\varepsilon }}_{\delta }{\dot{\hat{\varepsilon }}}_{\delta }\\ =s\left[\dot{e}+\frac{p}{\beta q}{\dot{e}}^{p/q-1}\left(f\left(x\right)+bu+\delta -{\ddot{x}}_{d}\right)\right]-\frac{1}{\eta_{1}}\tilde{W}\dot{\hat{W}}-\frac{1}{\eta_{2}}\tilde{V}\dot{\hat{V}}-\frac{1}{\eta_{3}}{\tilde{\varepsilon }}_{f\left(x\right)}{\dot{\hat{\varepsilon }}}_{f\left(x\right)}-\frac{1}{\eta_{4}}{\tilde{\varepsilon }}_{\delta }{\dot{\hat{\varepsilon }}}_{\delta }\\ =s\left[\dot{e}+\frac{p}{\beta q}{\dot{e}}^{p/q-1}\left(-\frac{\beta q}{p}{\dot{e}}^{2-p/q}+f\left(x\right)-\hat{f}\left(x\right)+\delta -\hat{\delta }-hsgn\left(s\right)\right)\right]-\frac{1}{\eta_{1}}\tilde{W}\dot{\hat{W}}-\frac{1}{\eta_{2}}\tilde{V}\dot{\hat{V}}-\frac{1}{\eta_{3}}{\tilde{\varepsilon }}_{f\left(x\right)}{\dot{\hat{\varepsilon }}}_{f\left(x\right)}-\frac{1}{\eta_{4}}{\tilde{\varepsilon }}_{\delta }{\dot{\hat{\varepsilon }}}_{\delta }\\ =-\frac{ps}{\beta q}{\dot{e}}^{p/q-1}h\left|s\right|+\frac{ps}{\beta q}{\dot{e}}^{p/q-1}\tilde{W}\phi +\frac{ps}{\beta q}{\dot{e}}^{p/q-1}\tilde{V}\phi +\frac{ps}{\beta q}{\dot{e}}^{p/q-1}{\tilde{\varepsilon }}_{f\left(x\right)}+\frac{ps}{\beta q}{\dot{e}}^{p/q-1}{\tilde{\varepsilon }}_{\delta }-\frac{1}{\eta_{1}}\tilde{W}\dot{\hat{W}}-\frac{1}{\eta_{2}}\tilde{V}\dot{\hat{V}}-\frac{1}{\eta_{3}}{\tilde{\varepsilon }}_{f\left(x\right)}{\dot{\hat{\varepsilon }}}_{f\left(x\right)}-\frac{1}{\eta_{4}}{\tilde{\varepsilon }}_{\delta }{\dot{\hat{\varepsilon }}}_{\delta }\text{.} \end{array}$$

We set the adaptive update law of the weights as

(32) $$\begin{array}{c} \dot{\hat{W}}=-\eta_{1}\frac{ps}{\beta q}{\dot{e}}^{\left(p/q\right)-1}\phi \text{,}\\ \dot{\hat{V}}=-\eta_{2}\frac{ps}{\beta q}{\dot{e}}^{\left(p/q\right)-1}\phi \text{,}\\ {\dot{\hat{\varepsilon }}}_{f\left(x\right)}=-\eta_{3}\frac{ps}{\beta q}{\dot{e}}^{\left(p/q\right)-1}\phi \text{,}\\ {\dot{\hat{\varepsilon }}}_{\delta }=-\eta_{4}\frac{ps}{\beta q}{\dot{e}}^{\left(p/q\right)-1}\phi \text{.} \end{array}$$

We substitute the adaptive update law of the weights into the derivative of the Lyapunov function as follows:

(33) $$\begin{array}{c} \dot{L}=-\frac{p}{\beta q}{\dot{e}}^{\left(p/q\right)-1}h\left|s\right|\leq 0\text{.} \end{array}$$

$\dot{L}$ becomes negative semidefinite. This implies that the trajectory reaches the sliding surface in finite time and remains on the sliding surface. Since $\dot{L}\leq 0$ , it is known that *L*(*t*) ≤ *L*(0), showing that $s\left(t\right),\tilde{W},\tilde{V},{\tilde{\varepsilon }}_{f\left(x\right)},{\tilde{\varepsilon }}_{\delta }$ is bounded. We define the following equation:

(34) $$\begin{array}{c} Z\left(t\right)=\frac{p}{2\beta q}{\dot{e}}^{\left(p/q\right)-1}h\left|s\right|\leq -\dot{L.} \end{array}$$

Then,

(35) $$\begin{array}{c} \int_{0}^{t}Z\left(\tau \right)d\tau \leq L\left(0\right)-L\left(t\right)\text{.} \end{array}$$

Since *L*(0) is bounded and *L*(*t*) is nonincreasingly bounded, it follows that

(36) $$\begin{array}{c} \lim_{t⟶\infty }\int_{0}^{t}Z\left(\tau \right)d\tau <\infty \text{.} \end{array}$$

*Z*(*t*) is uniformly continuous. Using Barbalat's lemma [11], the following results can be obtained:

(37) $$\begin{array}{c} \lim_{t⟶\infty }Z\left(t\right)=0\text{.} \end{array}$$

Thus, it can imply that $s\left(t\right),e,\dot{e}$ will converge to zero as *t*⟶*∞*. Therefore, the ENN-NTSMC-based control system guarantees the globally asymptotic stability of the tracking error in the presence of uncertainties, external disturbances, and faults.

The tracking error will reach the nonsingular terminal sliding surface in a finite time *t*_*r*_ which satisfies

(38) $$\begin{array}{c} t_{r}\leq \frac{\left|s\left(0\right)\right|}{\zeta }\text{,} \end{array}$$

where *ζ* is a positive constant.

Then, after entering the sliding mode surface, the finite convergence time can be obtained from equation (21) as

(39) $$\begin{array}{c} t_{f}=\frac{{\left|e\left(t_{r}\right)\right|}^{1-\left(p/q\right)}}{\beta \left(1-q/p\right)}\text{.} \end{array}$$

## 4. Design of Longitudinal DLC-ALCS under Fault Condition

**Figure 6:**
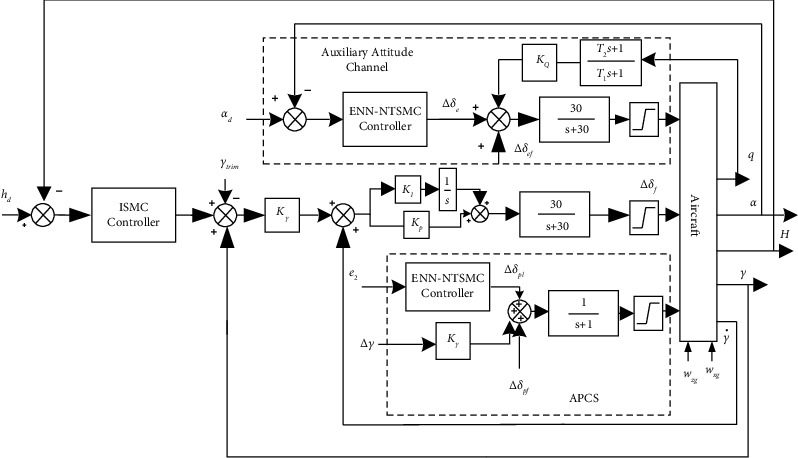
Structure diagram of the direct lift automatic landing fault-tolerant control system based on ENN-NTSMC.

The longitudinal DLC-ACLS is shown in Figure 6, mainly composed of the longitudinal guidance law, auxiliary attitude channel, and approach power compensator system (APCS). The longitudinal guidance law can deliver instructions and convert height variations to trajectory angle deviations. The function of the auxiliary attitude channel is to balance the pitch moment with the flaps, maintain the constant AOA, and increase the damping by feeding back the pitch angle rate. The function of the APCS is to automatically adjust the throttle and control the approach velocity of the aircraft to keep it constant to ensure the stability of the aircraft's long-term motion. In this section, a novel fault-tolerant control method based on ENN-NTSMC technology is designed, which is applied to the auxiliary attitude channel and APCS to ensure accurate control of the DLC-ACLS trajectory in the event of an unknown fault state. The design of the longitudinal guidance law is based on the integral sliding mode.

### 4.1. Auxiliary Attitude Channel Controller Design Scheme

The design of the auxiliary attitude channel controller, with the flaps to balance the pitch moment, maintains a constant AOA. Equation (40) describes the relationship between the AOA, the pitch angle, and the trajectory angle in a calm atmosphere.

(40) $$\begin{array}{c} \theta =\alpha +\gamma \text{.} \end{array}$$

Substituting equation (40) into equation (1) yields the following equation:

(41) $$\begin{array}{c} \dot{\alpha }=\frac{L+P\;\sin\;\alpha }{mV}-\frac{g}{V}\cos\;\gamma +\dot{\theta }\text{.} \end{array}$$

The linearized expansion of equation (41) at the reference point can be expressed in its specific form as the following equation:

(42) $$\begin{array}{c} ∆\dot{\alpha }=-\frac{1}{mV}∆L-\frac{∆P\;\cos\alpha_{*}-P_{*}∆\alpha \;\sin\alpha_{*}}{mV}-\frac{1}{V}g∆\gamma \;\sin\;\gamma +\Delta \dot{\theta }\text{.} \end{array}$$

The lift force can be linearized and expressed as

(43) $$\begin{array}{c} ∆L={L_{v}}_{*}∆v+{L_{\alpha }}_{*}∆\alpha +{L_{\delta e}}_{*}∆\delta_{e}+{L_{\delta c}}_{*}∆\delta_{c}+{L_{f}}_{*}∆\delta_{f}\text{.} \end{array}$$

Substituting equation (43) into equation (42), equation (42) becomes

(44) $$\begin{array}{c} ∆\dot{\alpha }=-\frac{1}{mV}\left[{L_{v}}_{*}∆v+{L_{\alpha }}_{*}∆\alpha +{L_{\delta e}}_{*}∆\delta_{e}+{L_{\delta c}}_{*}∆\delta_{c}+{L_{f}}_{*}∆\delta_{f}\right]\text{,}\\ -\frac{∆P\;\cos\alpha_{*}-P_{*}∆\alpha \;\sin\alpha_{*}}{mV}-\frac{1}{V}g∆\gamma \;\sin\;\gamma +∆\dot{\theta }\text{,} \end{array}$$

where

(45) $$\begin{array}{c} f_{1}=-\frac{1}{mV}\left[{L_{v}}_{*}∆v+{L_{\alpha }}_{*}∆\alpha +{L_{\delta c}}_{*}∆\delta_{c}+{L_{f}}_{*}∆\delta_{f}\right]\\ -\frac{∆P\;\cos\alpha_{*}-P_{*}∆\alpha \;\sin\alpha_{*}}{mV}-\frac{1}{V}g∆\gamma \;\sin\;\gamma +∆\dot{\theta }\text{,}\\ b_{1}=-\frac{1}{mV}{L_{\delta e}}_{*}. \end{array}$$

The equation is the first-order equation of the AOA related to the elevator deflection, but in the auxiliary attitude channel, the role of the elevator actuator must also be considered. In this article, the transfer function of the elevator is a first-order inertial link, so equation (44) becomes the second-order form, and equation (46) is as follows:

(46) $$\begin{array}{c} ∆\ddot{\alpha }=f_{1}+b_{1}∆\delta_{e}\text{.} \end{array}$$

When the elevator fails, the equation becomes

(47) $$\begin{array}{c} \ddot{\alpha }=f_{1}+b∆\delta_{e}+\delta_{1}\text{,} \end{array}$$

where *δ*_1_=*b*_1_∆*δ*_*ef*_, the error is defined as *e*_1_=*α* − *α*_*d*_, *α*_*d*_ is the desired AOA reference input, and the sliding mode surface is defined as follows:

(48) $$\begin{array}{c} s_{1}=e_{1}+\frac{1}{\lambda_{1}}{{\dot{e}}_{1}}^{\left(p_{1}/q_{1}\right)}\text{.} \end{array}$$

Based on the analysis for conventional second-order fault systems, the elevator control law is

(49) $$\begin{array}{c} ∆\delta_{e}=b_{1}^{-1}\left(-\frac{\beta_{1}q_{1}}{p_{1}}{\dot{e}}_{1}^{2-\left(p_{1}/q_{1}\right)}-{\hat{f}}_{1}\left(x\right)+{\ddot{\alpha }}_{d}-\eta_{1}sgn\left(s\right)-{\hat{\delta }}_{1}\right)\text{,} \end{array}$$

where the values of *β*_1_, *q*_1_, *p*_1_ and *η*_1_ are derived by simulation debugging.

### 4.2. Approach Power Compensator System Controller Design Scheme

The approach power compensator system (APCS) uses a velocity hold because the velocity control of the automatic throttle can be well maintained under the DLC. Its function is to automatically adjust the throttle and control the aircraft's approach velocity to keep it constant to ensure the aircraft's long-term motion stability.

Equation (3) is linearized and expanded at the reference point, and its specific form can be expressed as the following equation:

(50) $$\begin{array}{c} ∆\dot{V}=\frac{∆P\;\cos\alpha_{*}-P_{*}∆\alpha \;\sin\alpha_{*}}{m}-\frac{∆D}{m}-g∆\gamma \;\cos\gamma_{*}\text{.} \end{array}$$

The amount of change in thrust ∆*P* relative to the throttle deflection angle ∆*δ*_*p*_ can be expressed in the form of the following equation:

(51) $$\begin{array}{c} ∆P={\left(\frac{\partial P}{\partial \delta_{p}}\right)}_{*}∆\delta_{p}\text{.} \end{array}$$

Taking equations (51) to (50) yields

(52) $$\begin{array}{c} ∆\dot{V}=\frac{\cos\alpha_{*}}{m}{\left(\frac{\partial P}{\partial \delta_{p}}\right)}_{*}∆\delta_{p}-\frac{P_{*}∆\alpha \;\sin\alpha_{*}}{m}-\frac{∆D}{m}-g∆\gamma \;\cos\gamma_{*}\text{,} \end{array}$$

where

(53) $$\begin{array}{c} \left\{\begin{array}{c} b_{2}=\frac{\cos\alpha_{*}}{m}{\left(\frac{\partial P}{\partial \delta_{p}}\right)}_{*}\text{,}\\ f_{2}=-\frac{P_{*}∆\alpha \;\sin\alpha_{*}}{m}-\frac{∆D}{m}-g∆\gamma \;\cos\gamma_{*}\text{.} \end{array}\right. \end{array}$$

Considering that the throttle stick actuator transfer function used in the simulation is the same as that of the elevator actuator, it is also a first-order inertial link, so equation (52) can be converted into the following equation:

(54) $$\begin{array}{c} ∆\ddot{V}=b_{2}∆\delta_{pl}+f_{2}\text{.} \end{array}$$

When the throttle fails, the equation (54) becomes

(55) $$\begin{array}{c} ∆\ddot{V}=b_{2}∆\delta_{pl}+f_{2}+\delta_{2}\text{,} \end{array}$$

where *δ*_2_=*b*∆*δ*_*pf*_. The error is defined as *e*_2_=*V* − *V*_*d*_, *V*_*d*_ is the desired airspeed reference input, and the sliding mode surface is defined as follows:

(56) $$\begin{array}{c} s_{2}=e_{2}+\frac{1}{\lambda_{2}}{\dot{e}}_{2}^{\left(p_{2}/q_{2}\right)}\text{.} \end{array}$$

Based on the analysis for conventional second-order fault systems, the thrust control law is

(57) $$\begin{array}{c} \delta_{pl}=b_{2}^{-1}\left(-\frac{\beta_{2}q_{2}}{p_{2}}{\dot{e}}_{2}^{2-\left(p_{2}/q_{2}\right)}-{\hat{f}}_{2}\left(x\right)+{\ddot{V}}_{d}-\eta_{2}sgn\left(s\right)-{\hat{\delta }}_{2}\right)\text{,} \end{array}$$

where the values of *p*_2_, *q*_2_, *β*_2_, and *η*_2_ are obtained by simulation debugging.

### 4.3. The Longitudinal Guidance Law Control Scheme Controller Design Scheme

Due to the interference of the carrier air-wake on the landing of the carrier-based aircraft, the conventional control system has poor robustness in the face of various random disturbances. Moreover, whether the landing is guided by the carrier-based radar or the satellite, the measured altitude information contains various high-frequency noise signals. There are also noise signals in the interaction of the aircraft data chain, which will adversely affect the accurate control of the actual altitude. Therefore, the longitudinal guidance law can use the integral sliding mode control strategy to suppress disturbance effectively.

Equation (3) describes the relationship between the altitude change rate and the track angle, which can be linearly expanded at the reference point to obtain the following formula:

(58) $$\begin{array}{c} \frac{d∆h}{dt}=\sin\gamma_{*}∆v+v_{*}\cos\gamma_{*}∆\gamma \text{.} \end{array}$$

The equation leads to

(59) $$\begin{array}{c} ∆\dot{h}=f+b∆\gamma \text{,} \end{array}$$

where

(60) $$\begin{array}{c} y=x_{1}=∆hu=∆\gamma \text{.} \end{array}$$

Equation (59) can be transformed into the following formula:

(61) $$\begin{array}{c} {\dot{x}}_{1}=f+bu\text{.} \end{array}$$

The height control loop is a first-order system, and the integral sliding mode controller (ISMC) can be designed to achieve the control objective. This method can deal well with the random changes of external disturbances and various noise disturbances during the landing process and achieve more accurate landing accuracy. The following design and analysis of the ISMC of the longitudinal guiding law are given.

The error is defined as *e*=*h* − *h*_*d*_, *h*_*d*_ is the desired glide height, and the sliding surface is defined as follows:

(62) $$\begin{array}{c} s=e+c_{1}\int e\text{.} \end{array}$$

The derivative of the sliding mode function is

(63) $$\begin{array}{c} \dot{s}=\dot{e}+ce\\ =\dot{h}-h_{d}+ce\\ =f\left(x\right)+bu+\delta -h_{d}+ce\text{.} \end{array}$$

The Lyapunov function is chosen as

(64) $$\begin{array}{c} V=\frac{1}{2}s^{2}\text{.} \end{array}$$

The derivative of the Lyapunov function is

(65) $$\begin{array}{c} \dot{V}=s\dot{s}=s\left(f\left(x\right)+bu-h_{d}+ce\right)\text{,} \end{array}$$

and then the control law is

(66) $$\begin{array}{c} u=-\frac{f\left(x\right)+h_{d}+ce+\eta_{3}sgn\left(s\right)}{b}\text{.} \end{array}$$

We substitute equation (66) into (65)

(67) $$\begin{array}{c} \dot{V}=-\eta_{3}\left|s\right|<0\text{.} \end{array}$$

System stability is guaranteed, and the longitudinal guide law control output is obtained, where the values of *c* and *η*_3_ are obtained by simulation debugging.

## 5. Simulation Results of Landing with Actuator Faults

The initial trimmed states of the aircraft are chosen as the initial values of the state of the carrier aircraft are set as follows: *V*=70*m*/*s*, *α*=9.1° , and *θ*=5.6° . The fault-tolerance and tracking performance of the system are verified in a computer simulation environment. The command signal *H*_*d*_ is the ideal slide path with a track angle of −3.5°, and air-wake turbulence is added. The ENN has two neurons, nine neurons, and two neurons for the input layer, hidden layer, context layer, and output layer, respectively. To examine the role of the ENN-NTSMC system, a comparative simulation of the landing performance of the carrier-based aircraft under the basic controller and the fault-tolerant controller was performed, considering actuator failure. The matrices of the longitudinal linear small disturbance equation in equation (11) are listed as follows:

(68) $$\begin{array}{c} A=\left[\begin{array}{ccccc} -0.0673 & 1.07 & 0 & -9.792 & 0.000211\\ -0.00448 & -0.4225 & 1 & 0.0086 & 1.3\times 10^{-5}\\ 2.05\times 10^{-4} & 0.486 & -0.1598 & -0.00047 & 0\\ 0 & 0 & 1 & 0 & 0\\ -0.061 & -69.87 & 0 & 69.87 & 0 \end{array}\right]\text{,}\\ B=\left[\begin{array}{ccc} -0.02578 & -0.001197 & 0.1071\\ -0.001223 & -8.9\times 10^{-5} & -3.5\times 10^{-4}\\ -0.0212 & 0.00513 & 1.14\times 10^{-5}\\ 0 & 0 & 0\\ 0 & 0 & 0\\ 0\\ 6.84\times 10^{-4}\\ 0.00336\\ 0\\ 0 \end{array}\right]\text{,}\\ C=\left[\begin{array}{ccccc} 1 & 0 & 0 & 0 & 0\\ 0 & 1 & 0 & 0 & 0\\ 0 & 0 & 1 & 0 & 0\\ 0 & 0 & 0 & 1 & 0\\ 0 & 0 & 0 & 0 & 1\\ 4.57\times 10^{-4} & 0.0402 & 0 & 0 & -1.44\times 10^{-6}\\ 0 & -1 & 0 & 1 & 0 \end{array}\right]\text{,}\\ D=\left[\begin{array}{cccc} 0 & 0 & 0 & 0\\ 0 & 0 & 0 & 0\\ 0 & 0 & 0 & 0\\ 0 & 0 & 0 & 0\\ 0 & 0 & 0 & 0\\ 1.25\times 10^{-4} & 9.1\times 10^{-6} & 0 & -6.9\times 10^{-5}\\ 0 & 0 & 0 & 0 \end{array}\right]\text{,}\\ E=\left[\begin{array}{cc} 0.076 & -0.14\\ 0.004 & 0.006\\ 0.00019 & -0.007\\ 0 & 0\\ 0 & 0 \end{array}\right]\text{,}\\ F=\left[\begin{array}{cc} 0 & 0\\ 0 & 0\\ 0 & 0\\ 0 & 0\\ 0 & 0\\ -5.45\times 10^{-4} & -4.911\times 10^{-4}\\ 0 & 0 \end{array}\right]\text{.} \end{array}$$

### 5.1. Constant-Value Failure

**Figure 7:**
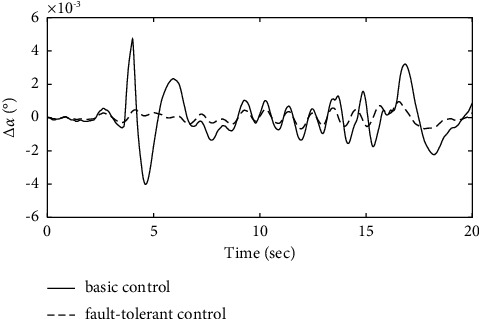
Comparison of the angle of the attack variation.

**Figure 8:**
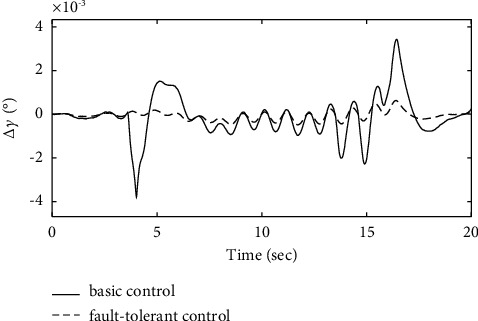
Comparison of the fight path angle variation.

**Figure 9:**
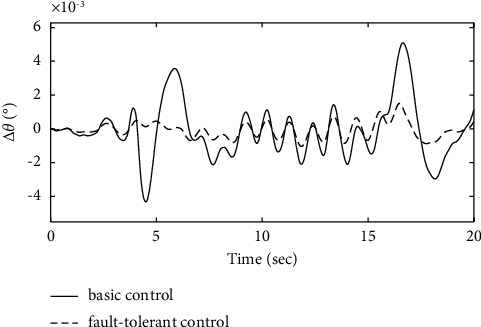
Comparison of the pitch angle variation.

**Figure 10:**
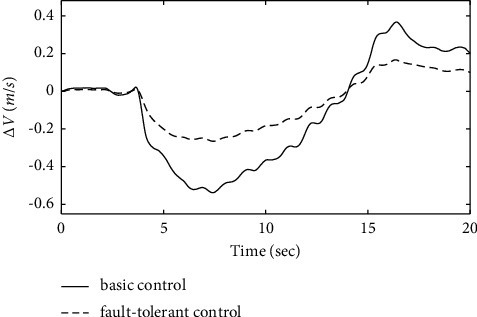
Comparison of the velocity variation.

**Figure 11:**
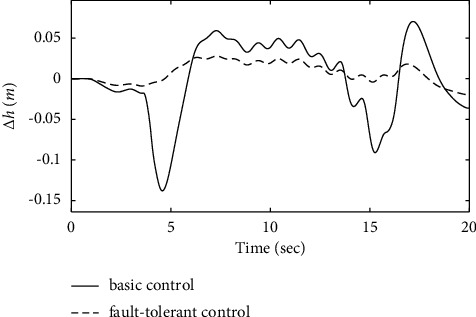
Comparison of the height variation.

**Figure 12:**
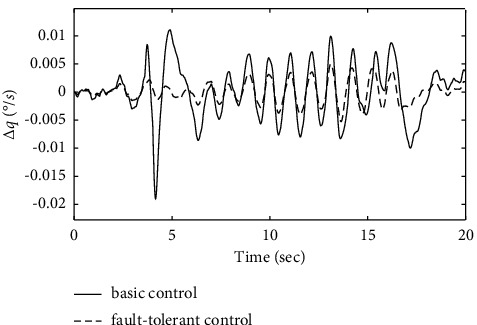
Comparison of the pitch rate variation.

**Figure 13:**
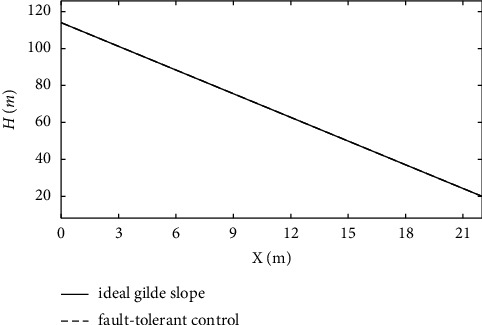
The variation curve of the glide path.

We consider the following constant-value fault in the actuator for simulation analysis, as shown in Figures 7–13. The engine fault *δ*_*pf*_=15° is introduced at *t*=3 *s*. The elevator fault *δ*_*ef*_=10° is introduced at *t*=3 *s*. The solid lines in Figures 7–13 represent the response curves of ∆*α*, ∆*θ*, ∆*V*, ∆*γ*, ∆*q*, ∆*h*, and *H* when only the NTSMC-based controller is used and the ENN estimator is not added.

From the simulation in Figures 7–12, it can be seen that the curves of ∆*α*, ∆*θ*, ∆*V*, ∆*γ*, ∆*q*, and∆*h* have a certain overshoot, when the aircraft suffers from engine and elevator failure, and the performance of the system is degraded, which is recovered after approximately 4 s of adjustment, but the curves still have obvious fluctuations. The dashed line represents the response curves of ∆*α*, ∆*θ*, ∆*V*, ∆*γ*, ∆*q*, ∆*h*, and *H* under the effect of the fault-tolerant control law added to the ENN estimator. When the actuator fault occurs, the ENN controller compensates for the system fault in time and accurately. To ensure that the system accurately tracks the state command, it achieves good tracking performance and robustness.  Figure 13 shows that the deviation between the actual flight path of the carrier-based aircraft and the ideal glide path is minimal. In summary, the fault-tolerant controller designed in this article ensures the tracking performance of the aircraft under the influence of a constant actuator fault.

### 5.2. Time-Varying Faults

**Figure 14:**
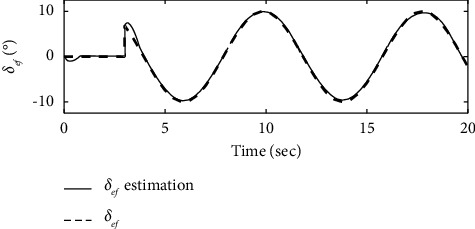
Elevator failure and its estimation.

**Figure 15:**
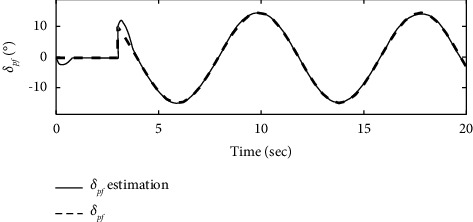
Throttle failure and its estimation.

**Figure 16:**
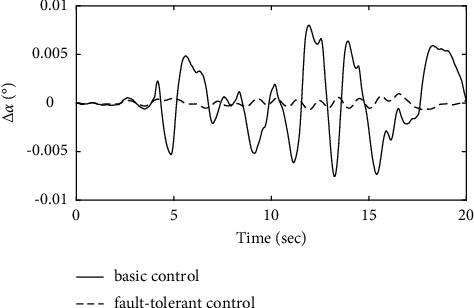
Comparison of the angle of the attack variation.

**Figure 17:**
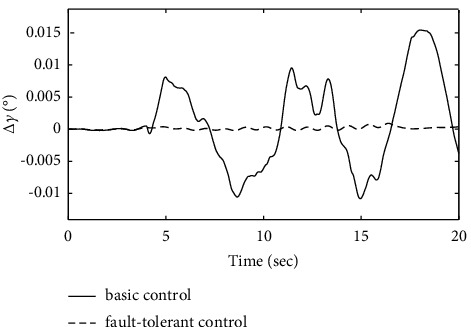
Comparison of the fight path angle variation.

**Figure 18:**
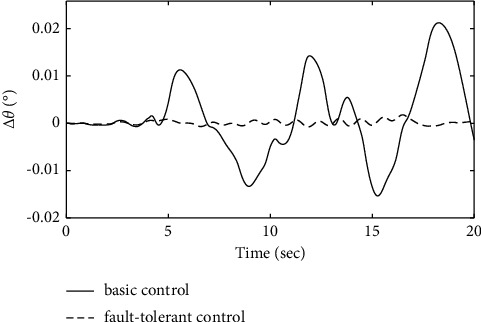
Comparison of the pitch angle variation.

**Figure 19:**
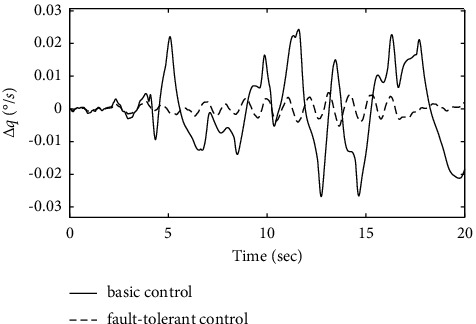
Comparison of the pitch rate variation.

**Figure 20:**
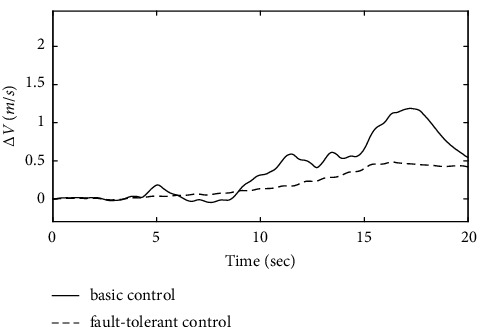
Comparison of the velocity variation.

To further verify the effectiveness of the ENN fault compensator designed in this article, a simulation analysis is carried out when the actuator has the following time-varying fault conditions, as shown in Figures 14–20. The thrust fault *δ*_*pf*_ = 15 sin *πt*/2.5(°) is introduced at *t* = 3*s*.The elevator fault *δ*_*ef*_ = 10 sin *πt*/2.5(°) is introduced at *t* = 3*s*. It is clear that the system state curves oscillate and do not achieve the desired control performance, and the system performance is worse than when a constant fault is encountered. Figures 14 and 15 show that the actuator failure is estimated accurately by using ENN.

From Figure 16, it can be seen that the AOA in the basic control strategy changes significantly after the introduction of the actuator failure, and the fault-tolerant control strategy designed in this article does not change significantly when the actuator fails, which proves that the auxiliary attitude channel with the fault-tolerant control strategy compensates effectively for elevator failure. After analysis, it is clear that the fault-tolerant control strategy designed in this article maintains a constant AOA of the carrier aircraft when the actuator fails.

Figures 17–19 show that through a comparative analysis with basic control strategies, it can be proven that the fault-tolerant control strategy designed in this article can effectively compensate for the actuator's failure, maintaining the changes in the trajectory angle and pitch attitude change in a small range. After analysis, the fault-tolerant control strategy designed in this article still has good robustness when actuator failure occurs.

Figure 20 shows that the approach airspeed change curve in the basic control strategy changes significantly after the introduction of the actuator time-varying fault. The fault-tolerant control strategy designed in this article does not change the approach airspeed significantly when the actuator fails, which proves that the power compensation system using the fault-tolerant control strategy can handle the throttle lever in time and effectively when the fault occurs. The fault-tolerant control strategy designed in this article can still maintain the stability of the approach speed of the ship when the actuator fails.

**Figure 21:**
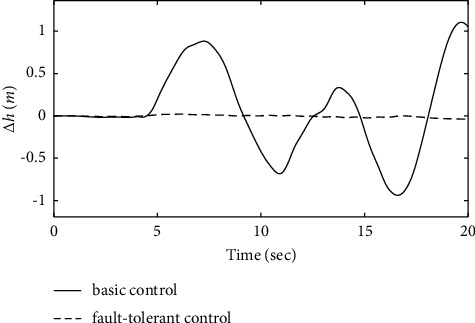
Comparison of the height variation.

**Figure 22:**
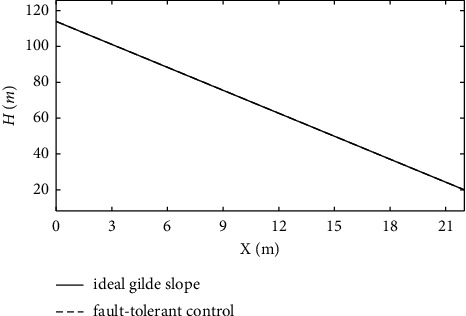
The variation curve of the glide path.

From Figure 21, it can be seen that the variation of altitude deviation in the fault-tolerant control strategy is slight compared with the basic control strategy. The analysis shows that the fault-tolerant control strategy still has good track-tracking capability in the fault state.  Figure 22 shows that the carrier-based aircraft can accurately track the ideal glide path. It can be seen from the calculation that the landing performance of the system under the action of the fault-tolerant control law meets the requirements of a safe landing, ensuring landing success.

## 6. Summary

This article studies the fault-tolerant technology of carrier-based aircraft, and a new fault-tolerant control method is proposed to optimize the longitudinal DLC-ALCS. First, the NTSMC-based control method is used as the basic controller to suppress the air-wake disturbance and solve the problem of accurate control of the flight trajectory. Then, the ENN estimator is introduced to compensate for the system failure caused by actuator failure, achieve the goal of robust fault-tolerant control, and test the effectiveness of this method under different types of actuator failures. The final simulation results show that with the basic controller, the system performance changes when the carrier-based aircraft system faults and the desired landing state is not achieved. However, under the fault-tolerant control of the ENN fault compensator, even if the actuator encounters a fault, the carrier-based aircraft system performance recovers quickly. It has strong robustness and fault-tolerant ability and realizes precise control of the landing trajectory. The performance index meets the safe landing requirements.

## Data Availability

The datasets used in this paper are available from the corresponding author upon request.
